# On the Versatility of Nanozeolite Linde Type L for Biomedical Applications: Zirconium-89 Radiolabeling and In Vivo Positron Emission Tomography Study

**DOI:** 10.1021/acsami.2c03841

**Published:** 2022-07-13

**Authors:** Sara Lacerda, Wuyuan Zhang, Rafael T. M. de Rosales, Isidro Da Silva, Julien Sobilo, Stéphanie Lerondel, Éva Tóth, Kristina Djanashvili

**Affiliations:** † Centre de Biophysique Moléculaire, CNRS UPR4301, Rue Charles Sadron, Orléans 45071 Cedex 2, France; ‡ Department of Biotechnology, Delft University of Technology, Van der Maasweg 9, Delft 2629 HZ, The Netherlands; § School of Biomedical Engineering & Imaging Sciences, St Thomas’ Hospital, King’s College London, London SE1 7EH, U.K.; ∥ CEMHTI, CNRS UPR3079, Université d’Orléans, Orléans 45071, France; ⊥ Centre d’Imagerie du petit Animal, PHENOMIN-TAAM, CNRS UAR44, Orléans F-45071, France; # Le Studium, Loire Valley Institute for Advanced Studies, 1 Rue Dupanloup, Orléans 45000, France

**Keywords:** nanozeolites, multimodal imaging, radiopharmaceuticals, positron emission tomography (PET), radiolabeling, zirconium-89

## Abstract

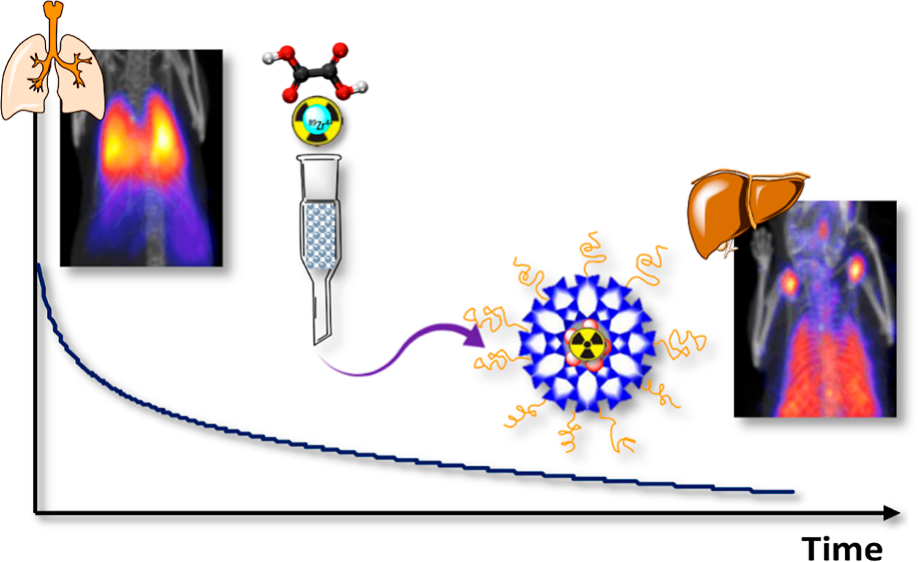

Porous materials, such as zeolites, have great potential for biomedical applications, thanks to their ability to accommodate positively charged metal-ions and their facile surface functionalization. Although the latter aspect is important to endow the nanoparticles with chemical/colloidal stability and desired biological properties, the possibility for simple ion-exchange enables easy switching between imaging modalities and/or combination with therapy, depending on the envisioned application. In this study, the nanozeolite Linde type L (LTL) with already confirmed magnetic resonance imaging properties, generated by the paramagnetic gadolinium (Gd^III^) in the inner cavities, was successfully radiolabeled with a positron emission tomography (PET)-tracer zirconium-89 (^89^Zr). Thereby, exploiting ^89^Zr-chloride resulted in a slightly higher radiolabeling in the inner cavities compared to the commonly used ^89^Zr-oxalate, which apparently remained on the surface of LTL. Intravenous injection of PEGylated ^89^Zr/Gd^III^-LTL in healthy mice allowed for PET–computed tomography evaluation, revealing initial lung uptake followed by gradual migration of LTL to the liver and spleen. Ex vivo biodistribution confirmed the in vivo stability and integrity of the proposed multimodal probe by demonstrating the original metal/Si ratio being preserved in the organs. These findings reveal beneficial biological behavior of the nanozeolite LTL and hence open the door for follow-up theranostic studies by exploiting the immense variety of metal-based radioisotopes.

## 1 Introduction

Technological advances in diagnostic imaging have generated a wealth of research on the design of imaging probes for the visualization of various physiological and pathological processes in the human body. The choice of the imaging modality is mainly conditioned by the clinical problem, while selection of the imaging agents relies on their physical and chemical properties, as well as their biological behavior. Among the different diagnostic techniques used in the clinic, positron emission tomography (PET) is known as a powerful, highly sensitive modality applied for functional imaging of (patho)physiological processes using picomolar amounts of radiotracers. Thereby, the choice of radionuclides and their carriers determines the biological fate of the probes.

As most of the existing positron-emitting radioisotopes are not suitable for PET due to complicated radiochemistry, poor availability, or high production costs, the focus of the research on possible PET tracers is driven mainly by these factors. Another major limitation is the short half-lives of the routinely applied PET tracers, such as ^18^F (*t*_1/2_ = 111 min) and ^11^C (*t*_1/2_ = 20.4 min), which do not match the long pharmacokinetic parameters of biological processes that might be of importance for certain pathologies.^1^ Another drawback is limited resolution of the technique,^2^ which needs to be complemented by high-resolution images that provide anatomical reference, for example, by coupling with computed tomography (CT) or magnetic resonance imaging (MRI). In this respect, a great deal of research is dedicated to the materials that combine different imaging modalities benefiting from the complimentary properties of each individual technique. Strategies providing easy combination of more than one imaging reporter offer great possibilities for preclinical research and personalized medicine.^3^ Thus, probes that allow for facile mix-and-match of functional moieties without changing the overall chemical properties and, most importantly, their biological behavior are very promising and deserve special attention.

This concept serves well the purpose of treatment planning and pretherapeutic assessment of probes by immuno-PET for which radiolabeled antibodies are used to identify whether a patient could benefit from a particular therapy based on antigen expression.^4^ An illustrative example of this strategy is the evaluation of the efficiency of ^90^Y-ibritumomab tiuxetan (^90^Y-Zevalin), anti-CD20 murine monoclonal antibody (MAb) for the treatment of B-cell non-Hodgkin’s lymphoma, using zirconium-89 (^89^Zr)-labeled surrogates.^5^ Injection of ^89^Zr-Zevalin in tumor-bearing nude mice resulted in very similar biodistribution, except for liver and bone accumulation, showing clear targeting of all known cancer sites in PET images. These findings indicate the feasibility of this method in a clinical setting to predict pharmacokinetics and biodistribution and to perform dosimetry of MAb-based radiotherapeutics.^6^

As the pharmacokinetics of antibodies is rather slow (3–4 days), long-lived β^+^-emitters are more adapted for antibody-based PET imaging. In this respect, transitional metal ^89^Zr is one of the promising radionuclides that has been receiving much attention over the last decade. With a long half-life of 78.4 h, it decays to the stable yttrium-89 (^89^Y) by positron emission (23%) and electron capture (77%) via the formation of the ultrashort-lived metastable ^89m^Y (*t*_1/2_ = 16 s). Moreover, the energies of positron emission of ^89^Zr are significantly low (*E*_max_ of 897 keV and *E*_ave_ of 397 keV), which results in PET images with high spatial resolution, when compared to ^124^I, a radionuclide with higher decay energy and comparable half-life (4.2 d).^7^ Furthermore, the energy of the ^89^Zr-photons produced during the accompanying gamma decay is 909 keV, which is convenient as it does not interfere with the energy of positrons (511 keV).

Among the materials suitable to assemble several functionalities, nanoparticles play a major role due to their high surface area available for conjugation with various vectors, high loading capacity, and the ability to penetrate the leaky blood vasculature surrounding neoplastic tissues.^8^ Radiolabeling of nanoparticles through chelate-free methodologies is an attractive strategy to avoid alteration of their pharmacokinetic properties.^9^ Thereby, silica-based nanoparticles are particularly interesting due to the possibility of intrinsic complexation of various metal ions without the need for additional ligands^10−12^ and good in vivo integrity.^13,14^ In our previous research, we have explored the potential of Linde type (LTL) nanozeolite (dimension: 40 × 20 nm), which is a porous material composed of an aluminosilicate framework organized in small and big channel-like cavities suitable for loading with metal ions.^15^ With a simple ion-exchange procedure, lanthanide ions (Ln^III^) can be placed into the big cavities, while the subsequent calcination of the material at 400–600 °C activates the irreversible migration of these ions into the small pores.^16^ This peculiarity was exploited to load the luminescent europium (Eu^III^) into the small cavities without access of water molecules, which is beneficial for optical imaging.^17^ In the second step, the paramagnetic gadolinium (Gd^III^) was added to the big channels, leading to coordination of six water molecules, which were found to be in a very fast prototropic exchange with bulk water.^18^ Consequently, the probe exhibited exceptionally high longitudinal (*r*_1_) and transverse (*r*_2_) relaxivities (paramagnetic water proton relaxation rate enhancement per mM Gd^III^). The MRI performance evaluated on phantoms revealed a pH-dependent behavior of the probe with a maximum relaxivity measured at pH 7. Noteworthy, conjugation of bulky polyethylene glycol (PEG) chains (<6 wt %) on the surface of these nanoparticles to prolong their blood-circulation times did not disturb the water-exchange process through the pores of Gd^III^-loaded LTL.^19^ These promising results, along with full characterization of the obtained materials and demonstrated absence of in vitro toxicity, have encouraged further in vivo studies to assess the biological behavior of the material by PET imaging using ^89^Zr-labeling.

Here, we present investigation on different methods to label Gd^III^-LTL with the ^89^Zr PET tracer and we demonstrate that the best results are achieved using ^89^Zr-chloride rather than the most common ^89^Zr-oxalate. ^89^Zr/Gd^III^-LTL was injected into healthy mice to evaluate the in vivo behavior of the probe by PET. The results evidence the suitability of this porous material for MR imaging in combination with radionuclide imaging and foresee its therapeutic potential after conjugation of the surface with appropriate targeting vectors.

## 2 Experimental Section

### 2.1 Radiolabeling of Gd^III^-LTL Using ^89^Zr-Oxalate

The radionuclide ^89^Zr was purchased from the BV Cyclotron VU, The Netherlands. The labeling was performed by the addition of 30 μL of solution of ^89^Zr in oxalic acid (1.0 MBq) into 1.0 mL of Gd^III^-LTL^17^ suspension (1.0 mg mL^–1^) followed by incubation at 45 °C for 1.5 h. The pH value of the suspension was determined to be between 3.0 and 4.0. After incubation, the mixture was centrifuged at 10.000 rpm for 5 min, the radioactivities of both the supernatant and precipitate were measured, and the labeling yield was calculated.

### 2.2 Radiolabeling of Gd^III^-LTL Using ^89^Zr-Chloride

The radionuclide ^89^Zr was produced at the CEMHTI cyclotron (Orléans, France), via irradiation of a commercial high-purity yttrium foil (Alfa, Aesar, 99.9%, 250 μm) by proton beam (12 MeV; 2 μA, 4 h), via the reaction ^89^Y(p,n)^89^Zr. After irradiation, the irradiated foil is solubilized in HCl 6 M, and the obtained solution is purified in a hydroxamate Zr resin (TrisKem, Bruz, France). The column was eluted four times with 2.5 mL of HCl 6 M to recover Y and then rinsed four times with 2.5 mL of Milli-Q water, and finally, the ^89^Zr was recovered in 1.5 mL of oxalic acid 0.05 M. Aliquots of the separated fractions were diluted, and their radionuclide purity was assessed by gamma spectrometry with an HPGe detector. For the data acquisition, the samples were placed at 5 cm from the crystal. The HPGe detector was calibrated in energy and efficiency for this liquid geometry with certified standard radioactive sources (Cerca, France). For activity measurements, γ-ray spectrum analysis software package Genie 2000 (Canberra, USA) was used.

^89^Zr-oxalate solution was further loaded onto an activated QMA Waters Sep-Pak C-18 cartridge (Waters Corporation, Milford, USA), which is a strong anion-exchange acrylic acid/acrylamide copolymer on silica-diol (surface functionality—[C(O)NH(CH_2_)_3_N(CH_3_)_3_]^+^ Cl^–^, pore size 300 Å, particle size 37–55 μm, ligand density 0.22 mmol/g ligand), prewashed with 6 mL of MeCN, 10 mL of 0.9% saline, and 10 mL of water. This column was eluted with 40 mL of water, and after this step, >99.9% of the ^89^Zr-activity remained on the cartridge. The ^89^Zr-chloride was then recovered by elution with 0.5 mL of HCl 1.0 M.

The labeling of Gd^III^-LTL was performed by the addition of 30 μL of solution of ^89^Zr-chloride in water (100 MBq) to 5 mg of Gd^III^-LTL, and the suspension was stirred at 45 °C for 1.5 h. The pH was kept between 3 and 4. In the following, the mixture was centrifuged at 10.000 rpm for 5 min. The radioactivity was measured in a Capintec CRC-15R gamma counter (Camberra, USA), and the labeling yield was calculated by dividing the activity remained in the supernatant by the total activity before centrifugation. The absence of unbound ^89^Zr in the supernatant was confirmed by radio-thin-layer chromatography (TLC) (*R*_f_ = 0; no spot at *R*_f_ of ^89^ZrCl_4_ solution) using silica gel IBF-2 Baker plates and citrate buffer as the mobile phase. The TLCs were measured on a miniGITA Star RadioTLC system (Elysia, Raytest, Belgium). In this TLC system, the free ^89^Zr has an *R*_f_ of 0.9. The radiolabeling procedure was performed in triplicate.

### 2.3 PEGylation of ^89^Zr–Gd^III^-LTL

The prepared ^89^Zr/Gd^III^-LTL (5 mg) was suspended in 2.5 mL of ethanol/water (3:2) solution containing 50 μL of 25% NH_4_OH. mPEG_2000_–silane (45 mg, 2.5 × 10^–5^ mol) was dissolved in 0.1 mL of H_2_O, and 40 μL of this solution was added to the prepared suspension of ^89^Zr/Gd^III^-LTL. The mixture was stirred at 50 °C for 90 min, centrifuged at 10.000 rpm, washed three times, and finally redissolved in 1 mL of either water or saline.

### 2.4 Characterization of Zr-Loaded LTL Nanoparticles and Stability Evaluations

X-ray diffraction patterns were measured using a Bruker AXS/D8 Advance diffractometer equipped with a Lynxeye detector and Co Kα radiation (λ = 1.78897 Å, 35 kV, 40 mA). The measurement range was from 5 to 70° 2θ with a step size of 0.02° in the continuous mode and an acquisition time of 0.5 s per step. Nitrogen adsorption/desorption isotherms were obtained with a TriStar II 3020 analyzer at 77 K after degassing the samples (powders) at 90 °C for 1 h and then at 250 °C overnight, after which the isotherms were measured over the relative pressure range (*P*/*P*_O_) from 0.01 to 0.991 and back. The surface area of LTL samples (*S*_BET_) and the total pore volume (*V*_total_) were determined with the Brunauer–Emmett–Teller (BET) equation. The external surface area (*S*_external_) was calculated using the *t*-plot statistical approach. Colloidal and metal-binding stability of LTL nanoparticles was evaluated under “cold” conditions. PEGylated and non-PEGylated Zr/Gd^III^-LTL nanoparticles were suspended in PBS and human serum at a concentration of 1 mg mL^–1^ and placed in an ultrasound bath for 0.5 h. After that, the suspensions were placed in glass tubes, kept without stirring at 37 °C, and photographed after 24 h and 1 week. Hydrodynamic sizes of the PEGylated nanoparticles in PBS and serum overtime were measured by dynamic light scattering (DLS) on a Zetasizer NanoZS (Malvern, UK) at 25 °C applying 4.0 mW, 633 nm He–Ne laser, and the 173° back-scatter mode. The diameters (*D*_H_) in nm are reported as number-weighted mean values obtained from three individual measurements. Evaluation of leaching of Gd^III^ and Zr^IV^ ions from PEGylated and non-PEGylated LTL-nanoparticles was performed on dispersions of these nanoparticles (1.5 mg mL^–1^) in PBS and human serum after incubation in a shaker at 37 °C for 24 h. After that, the nanoparticles were centrifuged, and the presence of the leaked metal ions in the supernatant was quantified by inductively coupled plasma–optical emission spectrometry (ICP–OES) (see the ICP protocol below). EDTA challenge study was performed by incubating 2.5 mg of both PEGylated and non-PEGylated Zr/Gd^III^-LTL-nanoparticles in 1 mL of 1 mM solution of ethylenediaminetetraacetic acid (EDTA) stirring the suspension at 37 °C. After 24 h, the samples were centrifuged, the supernatant was analyzed by ICP–OES for the presence of Zr^IV^ and Gd^III^, 1 mL of fresh medium was added, and the incubation was continued, repeating this procedure for another 6 days.

The stability of the ^89^Zr/Gd^III^-LTL probe was studied in the presence of different media: PBS (Dulbecco, pH 7.4, without Ca and Mg), saline (0.9% NaCl), and human serum. For this, 20 μL aliquots of the suspension were mixed with 200 μL of the medium, and the tubes were kept at room temperature and the complex stability followed by radio-TLC (0.3 μL aliquots). The studies were performed in triplicates.

### 2.5 Relaxometric Studies

The longitudinal and transverse relaxation times of all samples were measured on an Agilent 400-MR DD2 NMR spectrometer with a 5 mm One NMR probe. The samples were prepared by suspending 2.5 mg of LTL in 2.0 mL of Milli-Q water containing 0.5 wt % of xanthan gum as a stabilizer. Longitudinal relaxation times were measured with the inversion recovery method. Transverse relaxation times were measured with the Carr–Purcell–Meiboom–Gill (CPMG) pulse sequence in which the length of the spin echo train was varied. An echo time of 0.5 ms was applied for all measurements. The concentration of Gd was determined using the bulk magnetic susceptibility (BMS) method.^20^

### 2.6 MR Phantom Imaging

Experiments were conducted on a BioSpec 94/21, 9.4 T horizontal magnet (Bruker BioSpin, Wissembourg, France) provided with a BG060 gradient system (inner diameter of 60 mm and maximal strength of 950 mT m^–1^). *T*_1_-weighted MR images were acquired at 25 °C with the spin-echo sequence, TE = 29.9 ms, repetition time (TR) varying from 37 to 1500 ms, and resolution 137 × 137 mm^2^ with a matrix 256 × 256 in 16 min acquisition.

### 2.7 PET/CT Phantoms and Radioactivity Measurements

PET phantom images were acquired with a NanoPET-CT TM preclinical animal scanner (Mediso Ltd., Bioscan Inc.) with an acquisition time of 15 min. OSEM was used as the reconstruction method (pixel size 0.29 mm, axial thickness 0.585 mm, and 8 iterations). The CT images were acquired with 55 kVp tube voltage and 1.2 s exposure time in 360 projections. The images of two modalities (PET–CT) were fused using Invivoscope (Bioscan) software. The radioactivity of the obtained samples was measured with a dose calibrator (CRC-25R, Capintec, USA) or a gamma counter (1282 CompuGamma, LKB Wallac, Finland).

### 2.8 Preparation of the Probe for In Vivo Studies

After radiolabeling and PEGylation, 5 mg of ^89^Zr/Gd^III^-LTL–PEG was dissolved in 1 mL of saline from which 0.1 mL was taken, filled up to 0.5 mL of water and left overnight. The resulting suspension was divided for three injections of 0.150 μL and 6 MBq each (≈0.15 mg of the probe).

### 2.9 PET Imaging

BalbC-By female mice (8 week old) were bred in the specific pathogen-free (SPF) animal facility at TAAM-CNRS, Orléans, France. Mice were acclimated for 7 days in the laboratory before experimentation and were maintained in sterilized filter-stopped cages inside a controlled ventilated rack and had access to food and water ad libitum. All animal experiments were carried out in accordance with the guidelines for animal experiments and under permission number 19861, from the French “Ministère de l’Enseignement Supérieur, de la Recherche et de l’Innovation”.

About 6 MBq (5.97 ± 1.3 MBq) of ^89^Zr/Gd^III^-LTL–PEG was injected intravenously (tail vein, 150 μL) in three mice, and the mice were imaged at 4 min, 2 h, 18 h, 4 d, and 7 d postinjection in a Nano-PET eXplore Vista (Sedecal, Spain) coupled to a Nano-SPECT/CT scanner (Mediso). Mice were anesthetized by 2% isoflurane (Iso-Vet, Piramal Healthcare) and were placed on a thermostatically controlled heating pad (37 °C) during imaging. Images were acquired for 30 min and reconstructed using MMWKS image software (4.7). CT images were reconstructed with Invivoscope (InVicro, v2.0), and the PET data were analyzed with VivoQuant (InVicro, v2.5) dedicated programs. Images are plotted as standardized uptake values (SUVs), taking into account ^89^Zr decay. The uptake in the organs (namely, lungs, liver, and bone) was measured by plotting different region of interests (ROIs) and analyzing the signal intensities after correction for decay. The results are expressed as the percentage of the organ ROI radioactivity dose per total initial dose (in counts/s) per volume of tissue ROI (in mL), as %ID/mL mean ± SEM (*n* = 3).

### 2.10 Biodistribution Studies

(A) Under “PET conditions”: after the last imaging time (7 d), the three mice were sacrificed, and the organs of interest were harvested (lungs, liver, bone, spleen, and muscle). After full radioactive decay (10 half-lives), their Si content was determined by ICP–OES (see the detailed ICP–OES protocol below). (B) Under “MRI-conditions”: additional three mice were intravenously injected (tail vain) with 150 μL suspension of 1.5 mg of Gd^III^-LTL–PEG in saline. After 24 h, the mice were sacrificed, the organs of interest were harvested (lungs, liver, spleen, and kidney), and their Gd and Si content was determined by ICP–OES (see the detailed protocol below). The Gd and Si content of a sample of the stock solution has also been measured by ICP–OES.

### 2.11 ICP–OES Measurements

ICP–OES was performed on a Jobin Yvon ULTIMA2 spectrometer (Longjumeau, France). Standard solutions of Si, Gd, and Zr were prepared using a commercial multielement solution 1 for ICP (Sigma-Aldrich, France) in 5% HNO_3_. Gd-loaded LTL, labeled either with Zr-oxalate or Zr-chloride, was hydrothermally solubilized in HNO_3_ in a microwave oven. For the ex vivo evaluations, the organ samples were digested in concentrated HNO_3_ (65%) for 48 h at room temperature and subsequently for 18 h at 65 °C. All measurements were performed in triplicate; data are presented as mean ± SD (*n* = 3).

## 3 Results and Discussion

### 3.1 Radiolabeling of Nanozeolite LTL with ^89^Zr

Initially, the LTL nanoparticles were loaded with paramagnetic Gd^III^ following the previously published procedure.^17^ The quantitative encapsulation of Gd^III^ was justified based on the theoretical capacity of LTL to accommodate up to 6.4 wt % of metal-ions, knowing that 3.6 alkali ions per unit cell in the large channels are prone to ion-exchange under mild conditions.^21^ Taking into account the subsequent loading with Zr-atoms, for this study, the LTL was suspended in a solution containing only 2.7 wt % of GdCl_3_. The mixture was stirred for 30 min at room temperature and subsequently centrifuged/washed a couple of times to yield a powder that was used for radiolabeling.

**Figure 1:**
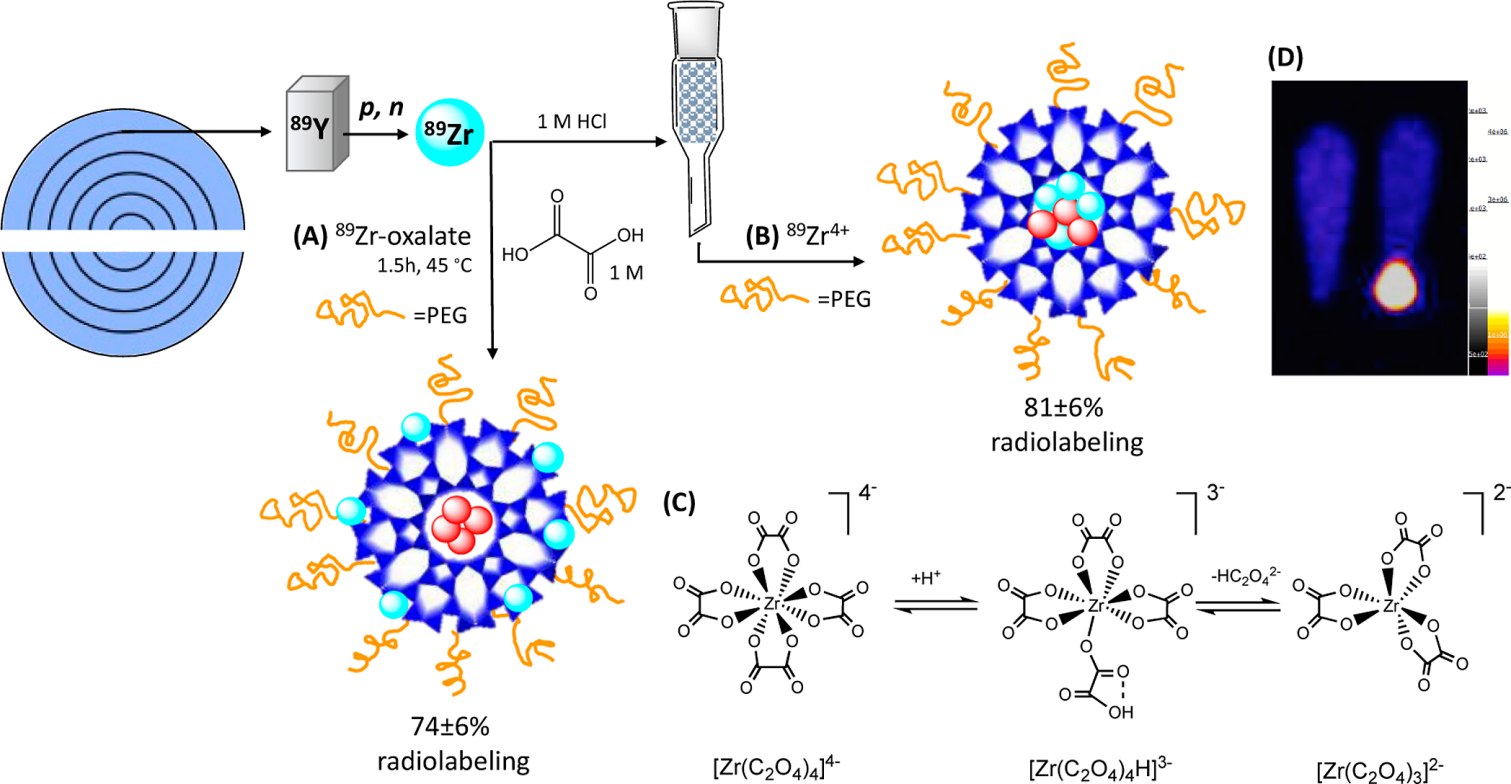
Schematic representation of the labeling of Gd^III^-LTL (Gd = red spheres) with cyclotron-produced ^89^Zr: (A) using ^89^Zr-oxalate; (B) after ion-exchange with chloride ions (thus ^89^ZrCl_4_); (C) structures of Zr-oxalate complexes existing in acidic solution; and (D) PET phantom images taken on centrifuged (left) and noncentrifuged (right) ^89^Zr/Gd^III^-LTL.

A typical production of ^89^Zr for preclinical applications takes place in small biomedical cyclotrons by bombarding solid ^89^Y-targets with low-energy proton beam (around 14 MeV) currents, resulting in ^89^Y(p,n)^89^Zr reaction.^22^ Increased beam energies (>17 MeV) might lead to higher radiochemical yields but are accompanied with the formation of long-lived byproducts, such as ^88^Zr (*t*_1/2_ = 83.4 d) and ^88^Y (*t*_1/2_ = 106.65 d) via the ^89^Y(p,2n)^88^Zr reaction.^22^ As these impurities represent a serious problem, the following steps of chelation of ^89^Zr in the final PET agent and separation strategies are under close attention of radiochemists.^23^ The proposed methods to isolate ^89^Zr from the target include extraction^24^ and chromatography by the exchange of either cations^25^ or anions.^26^ Typically, ion-exchange is applied for which the target is first dissolved in concentrated HCl, then the resulting solution is passed through a column, and finally, desorption of ^89^Zr is achieved by elution with a solution of oxalic acid in 1 M HCl.^27^ Based on literature reports on the highest efficiency of radiolabeling of MAb obtained with ^89^Zr-oxalate, this route was applied initially in this study (Figure 1A). In this method, Gd^III^-LTL was incubated at pH 3 and room temperature for 1.5 h in the presence of ^89^Zr-oxalate (1 MBq). The unlabeled material was removed by centrifugation, and a radiolabeling yield of 74 ± 6% was calculated, which corresponds to ca. 4.4 × 10^–13^ mol of ^89^Zr per gram of the solid material. This loading procedure, being the same as that for the Ln^III^ ions, follows a completely different mechanism in the case of ^89^Zr-oxalate. In solution, ^89^Zr-oxalate exists as [Zr(C_2_O_4_)_4_]^4–^, where the coordination number of Zr amounts to 8. Under acidic conditions, this oxalate complex partially dissociates to give six-coordinated [Zr(C_2_O_4_)_3_]^2–^,^28^ which creates space around Zr^IV^ for the binding of oxygen atoms of silanols on the surface of LTL.

The calculated diagonal dimension of [Zr(C_2_O_4_)_3_]^2–^ is 8.14 Å, which is larger than the pore opening of LTL (7.1 Å). Therefore, one can hypothesize that ^89^Zr-oxalate complexes coordinate strongly to the surface oxygens and do not enter the inner cavities. Furthermore, the negative charge of the Zr^IV^-oxalates is another factor to prevent them from entering the negatively charged AlO_4_^–^ framework of LTL. In this scenario, ^89^Zr-labeling occurs mainly on the surface of the LTL nanoparticles in the form of oxalate complexes, as depicted in Figure 1B.

Assuming possible accumulation of ^89^Zr on the nanozeolite surface, we have also explored a second method of radiolabeling, more adapted to label the interior space of the LTL. To achieve this, ^89^Zr-oxalate was converted to ^89^ZrCl_4_ by anion-exchange on a column composed of the acrylic acid/acrylamide copolymer conjugated to a silica-diol support,^29^ prior to incubation with Gd^III^-LTL (Figure 1C). To accomplish sufficient loading of Zr^IV^ into the framework, the mixture of zeolite with solution of ^89^ZrCl_4_ (100 MBq) was stirred for 1.5 h at 45 °C while maintaining the pH at 3. The final mixture was then centrifuged, and both the supernatant and the resulting ^89^Zr/Gd^III^-LTL resuspended in saline were analyzed by radio-TLC (Figure S1). This resulted in 81 ± 6% radiolabeling yield, which is only slightly higher compared to the first method. To directly demonstrate the successful radiolabeling, the ^89^Zr-labeled LTL sample was visualized in PET images before and after centrifugation (Figure 1D). It should be noted that the background signal in PET phantom images of these two (centrifuged and noncentrifuged) solutions appears visually similar; however, this is only the consequence of the image construction using the software for low-activity samples. At this point, it was impossible to ascertain if using the two methods, the Zr-ions are retained on the surface or enter the inner core. Moreover, there are virtually no spectroscopic or imaging techniques that could differentiate between the two populations of “cold” Zr-ions. The X-ray diffraction profiles of LTL loaded with Zr via both oxalate and chloride procedures correlate with the calculated patterns and with those of the preloaded Na-LTL (Figure S2), confirming the unaltered crystallinity, but are otherwise identical to each other.

Given the recent literature evidence of the ability of Zr-ions to enter the inner core of a NaX zeolite in the form of either ZrO^II^- or HZrO_2_^II^-ions, depending on the pH applied,^30^ it is reasonable to assume that at pH 3, the small positively charged ^89^Zr-ions can be loaded into the pores and coordinate to the framework next to the already incorporated Gd^III^, instead of being adsorbed on the surface. To investigate this, EDTA challenge experiments were included in the stability studies to elucidate possible ion-stripping from the surface of LTL nanoparticles. Furthermore, the BET analysis to examine the surface area and porosity of LTL after metal loading and the relaxivity study were proposed as elegant solutions to shade light at the position of Zr, resulting from different loading procedures (vide infra).

### 3.2 Stability and Functional Porosity of Zr/Gd^III^-LTL

**Figure 2:**
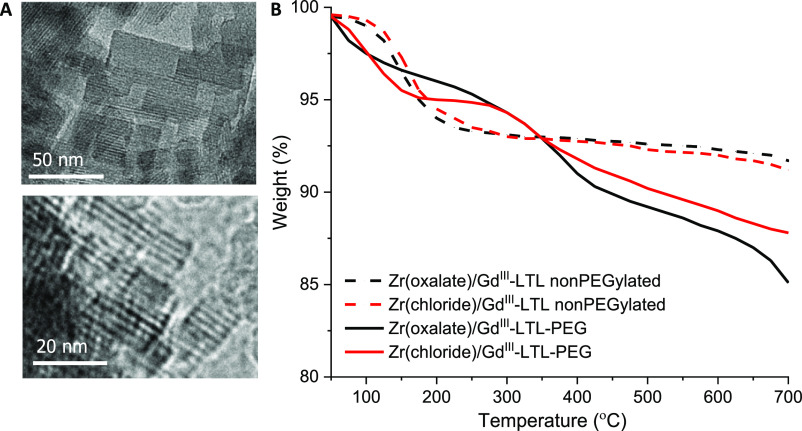
(A) TEM images of Gd^III^-LTL and (B) TGA profiles of Gd^III^-LTL loaded with Zr-chloride and Zr-oxalate before and after PEGylation.

Surface functionalization of nanoparticles is a common way to stabilize them prior to in vivo applications. In our previous study, we have shown that this strategy not only provides the nanoparticles with the necessary colloidal stability but also prevents the leakage of toxic ions loaded in the porous LTL crystals.^19^ Thus, in the current study, the PEGylation step was adapted to the radiolabeling procedure. For this, methoxy polyethylene glycol (mPEG_2000_) was conjugated with 3-aminopropyltrimethoxysilane (APTMS) prior to the attachment to the silanol-rich surface of LTL in order to reduce the PEGylation time. A mixture of zeolite, whose transmission electron microscopy (TEM) images are shown in Figure 2A, and an excess of mPEG_2000_–silane (1:5 w/w) were stirred at 50 °C for 90 min, followed by triple washing with phosphate buffer (PBS) and lyophilization of the material. The thermogravimetric analysis (TGA) of the samples produced under “cold” conditions using Zr in both oxalate and chloride forms confirmed successful PEGylation at 6 wt % (Figure 2B). Visual assessment of PEGylated Zr/Gd^III^-LTL nanoparticles incubated for up to 1 week at a concentration of 500 μg L^–1^ in either PBS or serum confirmed better colloidal stability compared to their non-PEGylated analogues (Figure S3). In addition, the hydrodynamic size of the nanoparticles measured by DLS appeared stable overtime with slightly higher values for the samples incubated in serum (around 160 nm) compared to those in PBS (around 130 nm) (Figure S4).

Stability of the systems was evaluated by incubating both PEGylated and non-PEGylated Zr(oxalate)/Gd^III^-LTL and Zr(chloride)/Gd^III^-LTL nanoparticles in PBS and human serum at 37 °C for 24 h and measuring the leakage of toxic metal-ions by ICP–OES. As shown in Table 1, the small leakage of Gd^III^ ions from Zr(oxalate)/Gd^III^-LTL and Zr(chloride)/Gd^III^-LTL nanoparticles in both media could be reduced upon PEGylation of their surface, as reported earlier for Gd^III^-LTL.^19^ The leakage of Zr^IV^ ions follows the same trend in PBS, while in serum, the difference between the two Zr-loading procedures becomes apparent for the non-PEGylated Zr(oxalate)/Gd^III^-LTL and Zr(chloride)/Gd^III^-LTL nanoparticles with 4.2 and 1.6 wt % released Zr^IV^ ions, respectively. This moderate leakage indicates a strong binding of this tetravalent metal-ion to the LTL framework, whether on the surface or in the inner pores. At the same time, the higher leakage of Zr^IV^ ions from Zr(oxalate)/Gd^III^-LTL in serum suggests competing interactions between the metal-ions and chelating moieties (e.g., proteins) that occur on the surface of nanoparticles. To investigate this phenomenon, a challenge study was performed, in which the nanoparticles were incubated with 1 mM solution of EDTA at 37 °C and the leakage of metal-ions was evaluated for 7 days (Table 1). Under these conditions, the internally loaded Gd^III^ ions showed the same insignificant leakage. On the other hand, the release of Zr^IV^ ions from both PEGylated and non-PEGylated Zr(oxalate)/Gd^III^-LTL was further increased to 5.8 and 15.8 wt %, respectively. Since no such effect was observed for Zr(chloride)/Gd^III^-LTL, the increased Zr-leakage from Zr(oxalate)/Gd^III^-LTL can be explained by the ion-stripping from the surface of LTL. Interestingly, 90% of Zr^IV^ ions have been removed from the surface of the non-PEGylated Zr(oxalate)/Gd^III^-LTL already within 24 h, while for the PEGylated analogue, the first-day Zr-leakage amounted 50% (Figure S5). This suggests that the bulky PEG chains not only prevent but also slow down competitive complexation with EDTA.

**Table 1:** Leakage of Zr^IV^ and Gd^III^ from the PEGylated (6 wt %) and non-PEGylated LTL-Nanoparticles at 37 °C in the Presence of PBS, Human Serum, and 1 mM Solution of EDTA as a Challenging Chelator^a^

	Zr(oxalate)/Gd^III^-LTL				Zr(chloride)/Gd^III^-LTL			
	Zr (wt %)		Gd (wt %)		Zr (wt %)		Gd (wt %)	
Medium	PEG	non-PEG	PEG	non-PEG	PEG	non-PEG	PEG	non-PEG
PBS^b^	0.7 ± 0.03	1.6 ± 0.3	0.4 ± 0.03	0.7 ± 0.02	0.5 ± 0.02	1.5 ± 0.2	0.7 ± 0.04	0.6 ± 0.07
serum^b^	0.8 ± 0.03	4.2 ± 1.1	0.7 ± 0.07	1.7 ± 0.3	0.7 ± 0.03	1.6 ± 0.5	0.7 ± 0.03	1.7 ± 0.3
EDTA^c^	5.8 ± 1.2	15.8 ± 2.1	0.5 ± 0.02	1.3 ± 0.6	0.9 ± 0.04	1.9 ± 0.3	0.6 ± 0.01	1.6 ± 0.4

a Data are obtained with ICP–OES; values are given as mean ± SD (*n* = 3).

b After 24 h.

c After 1 week.

**Figure 3:**
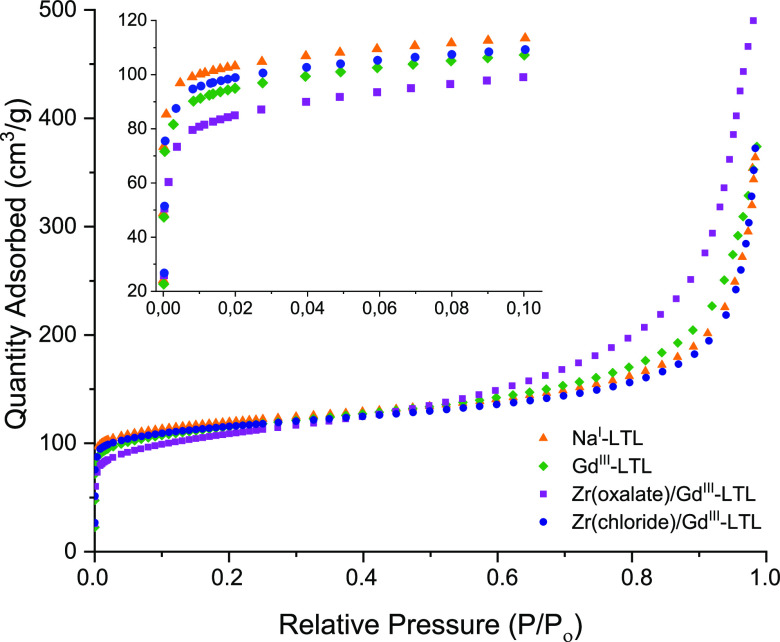
Nitrogen adsorption isotherms of LTL before (Na^I^) and after the loading with extra-framework Gd^III^ and Zr^IV^ via both oxalate and chloride procedures. Adsorption curve at low *P*/*P*_O_ is visualized in the inset.

To further demonstrate the impact of the extra-framework metal-ions on the functionality of the LTL nanocrystals, their porous properties were investigated by N_2_ adsorption–desorption isotherm analysis (Figures 3 and S6). The observed type I isotherms at low *P*/*P*_o_ (<0.1) confirm the preserved microporosity of all samples regardless of their metal loading (inset in Figure 3). At high *P*/*P*_o_ (>0.8), the isotherms exhibited type IV character, typical for the close packing of zeolite nanoparticles. The data calculated from the BET analysis show that the surface area (*S*_BET_), the external surface (*S*_external_), and the total pore volume (*V*_Total_) are comparable for the LTL before (Na^I^-LTL) and after the loading with Gd^III^ ions in the inner pores (Table 2). These values are also not very different for the Gd^III^-loaded LTL in which Zr^IV^ was added in the form of a chloride. A slightly lower *S*_BET_ (435 m^2^/m) measured for Zr(chloride)/Gd^III^-LTL compared to that of Gd^III^-LTL (445 m^2^/m) can be explained by the difference in the effective ionic radii of Gd^III^ (1.02 Å) and Zr^IV^ (0.72 Å), resulting in a smaller space occupied by the latter ion and thus a higher *S*_BET_. This phenomenon was already reported for the LTL nanocrystals loaded with small Li^I^ ions (0.76 Å) and compared with the native Na^I^ (1.02 Å).^31^ As for the external surfaces, the value of 190 m^2^/g obtained for Zr(oxalate)/Gd^III^-LTL being essentially higher than those calculated for all other samples, including Zr(chloride)/Gd^III^-LTL (103 m^2^/g), can be attributed to the increased roughness of the outer surface due to the presence of Zr-clusters, which consequently increases its *S*_external_. Moreover, the higher hysteresis in adsorption–desorption isotherms of Zr(oxalate)/Gd^III^-LTL (Figure S6) indicates irregular porosity that can be associated with the rough surface as a result of Zr-clustering on the surface. Finally, the lower values of *S*_BET_ and *V*_Total_ can be explained by the blockage of the pores, leading to inefficient adsorption.

**Table 2:** Porous Properties of LTL Nanocrystals before and after the Loading of Extra-framework Cations

sample	*S*_BET_ (m^2^/g)	*S*_external_ (m^2^/g)	*V*_total_ (cm^3^/g)
Na^I^–LTL	451	106	0.140
Gd^III^-LTL	445	107	0.136
Zr(chloride)/Gd^III^-LTL	435	103	0.154
Zr(oxalate)/Gd^III^-LTL	394	190	0.085

**Figure 4:**
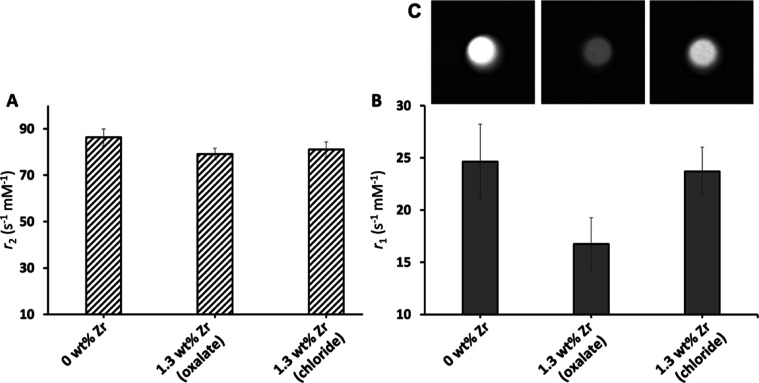
^1^H NMR relaxivities of PEGylated (6 wt %) LTL nanoparticles loaded with 2.7 wt % Gd^III^ and coloaded with 1.3 wt % Zr in either oxalate or chloride form, measured at 25 °C and a magnetic field strength of 9.4 T: (A) *r*_2_, (B) *r*_1_, and (C) *T*_1_-weighted spin-echo images (TE = 29.0 ms) of MR phantoms containing the corresponding Gd^III^-LTL suspended in 1% xanthan (0.3 mM of Gd), taken at 9.4 T and 25 °C.

The position of the extra-framework Zr-cation might influence the MRI performance of the final probe by interfering with the water-exchange process through the pores of LTL. In our previous study, we have already demonstrated both longitudinal and transverse relaxivities of Gd^III^-loaded LTL to be preserved, provided that the amount of PEG on the surface does not exceed 6 wt %. Here, we looked into possible alteration of relaxivities as a result of Zr-loading. Therefore, the effect of the two labeling methods was assessed by measuring both *r*_1_- and *r*_2_-relaxivities at 25 °C and 9.4 T. The results were compared with those of the native material PEGylated at 6 wt % and containing the same amount of Gd^III^ (2.7 wt %) (Figure 4). Although the *r*_2_-relaxivities were not affected by the Zr-loading procedure (Figure 4A), the *r*_1_-relaxivities, which rely on water exchange, showed a clear difference between the Zr-oxalate- and Zr-chloride-loaded samples (Figure 4B). Furthermore, the differences in signal intensities in *T*_1_-weighted MR images (Figure 4C) of the phantoms of Gd^III^-LTL loaded with 0 wt % Zr and 1.3 wt % of either Zr-oxalate or Zr-chloride confirm the effect of blocking of the pores by Zr-oxalate on the surface of LTL. Indeed, the *r*_1_-relaxivity of the sample prepared with Zr-oxalate is considerably lower, which is related to limitations in the water-exchange process between the Gd^III^ sites inside the pores and bulk water, most probably caused by the presence of bulky Zr^IV^-complexes on the surface of LTL in addition to already present PEG-chains. In contrast, there is no restriction of the water exchange for the sample loaded with Zr-chloride, and hence, it can be assumed that in this procedure, the Zr-ions enter the pores and do not block the water access. Considering the relaxivity difference as unambiguous evidence of the different Zr-loading methods, we extrapolate that “hot” samples should behave in similar way. In any case, it is clear that the position of extra-framework metal-ions in the nanocrystal will have an impact on the in vivo functionality of the probe, and this study reinforces the rationale for using the modified ^89^Zr-loading procedure to achieve efficient MRI performance and simultaneous radiolabeling.

### 3.3 Radiosynthesis and In Vivo Imaging

We have further evaluated the in vivo behavior of the Gd^III^-LTL probe, radiolabeled with ^89^ZrCl_4_, using the second procedure described above. Therefore, ^89^Zr-oxalate was first ion-exchanged with chloride, and the final solution (^89^ZrCl_4_, 100 MBq) was used for loading of Gd^III^-LTL, which was then PEGylated following the abovementioned procedure. The absence of the unbound ^89^Zr was confirmed by TLC by comparing an aliquot sample taken from the supernatant postcentrifugation of the ^89^Zr/Gd^III^-LTL with a sample of the ^89^ZrCl_4_ mother solution (Figure S1). Furthermore, the ^89^Zr/Gd^III^-LTL–PEG probe was found stable in the presence of different media up to 7 days (Figure S7).

**Figure 5:**
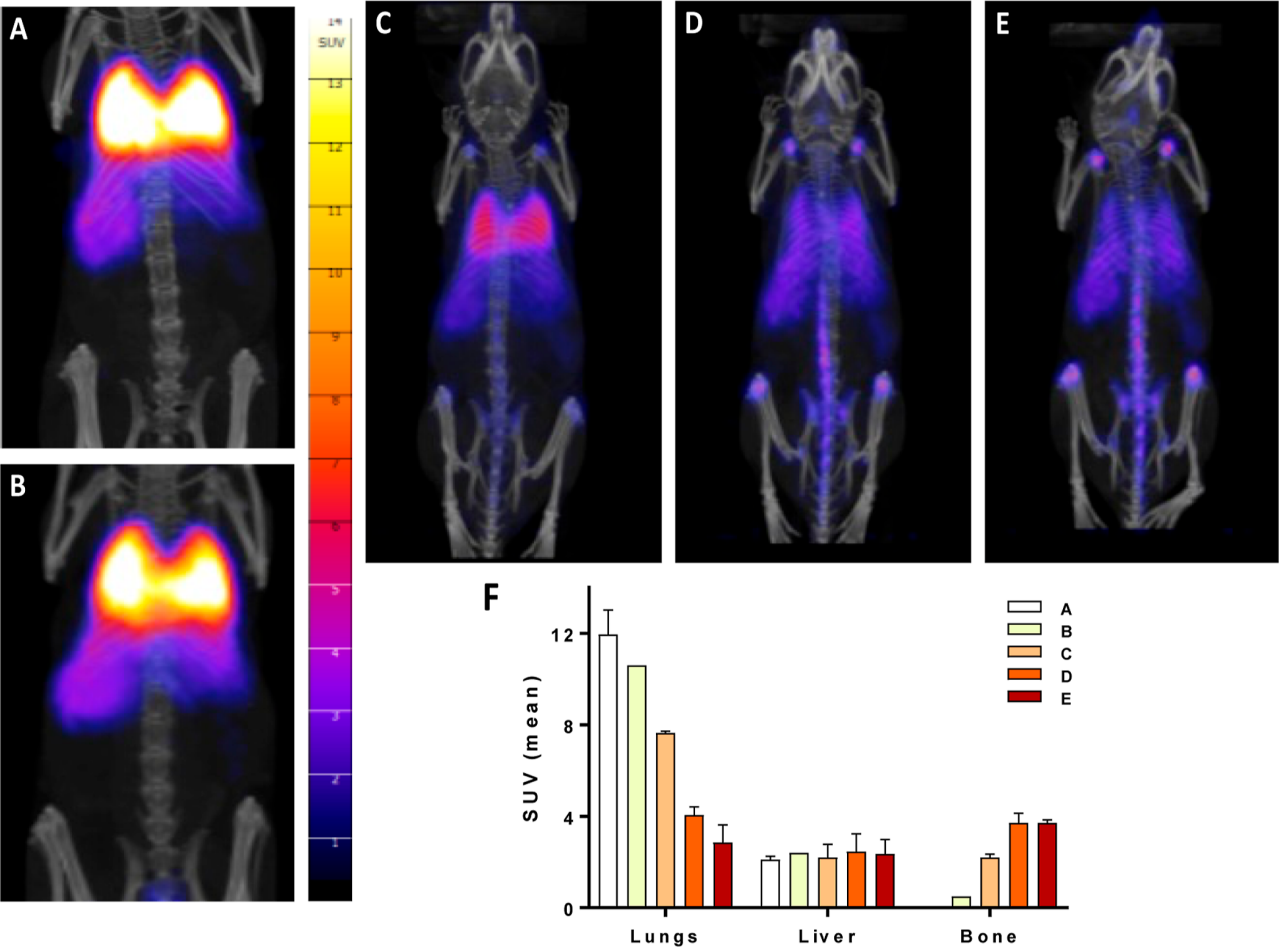
PET–CT images acquired at 4 min (A), 2 h (B), 18 h (C), 4 d (D), and 7 d (E) after intravenous injection of ^89^Zr/Gd^III^-LTL (6 MBq) in healthy mice. Images were corrected for activity decay; intensities of organ accumulations (F) are shown in SUVs (*n* = 3, mean ± SD).

**Figure 6:**
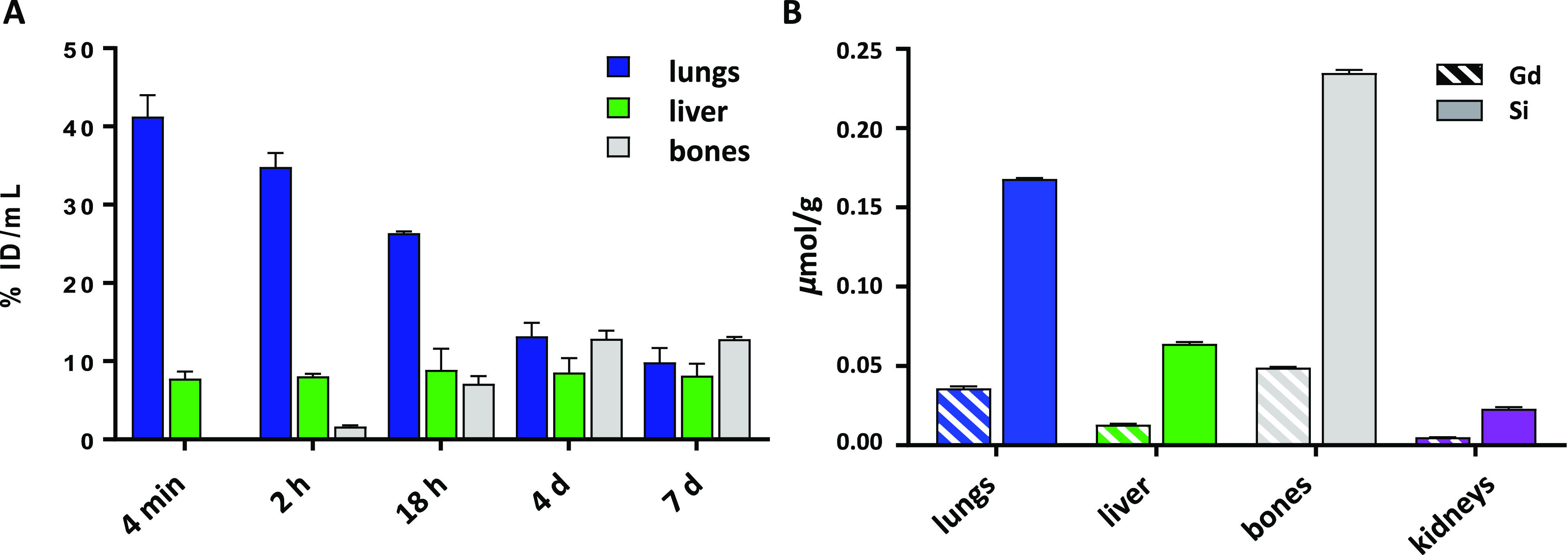
Biodistribution studies: (A) In vivo—calculated as the percentage of injected dose per volume (% ID/mL ± SEM, *n* = 3) from ROIs in the PET–CT images (Figure 5) and (B) ex vivo—calculated by ICP–OES under “MRI-conditions” by determining Gd- and Si-content. Animals were euthanized after 24 h p.i., and organs were excised and digested in HNO_3_ prior to the analysis; data are presented in μmol/g (±SEM, *n* = 3).

For the in vivo studies, three healthy mice were intravenously injected (caudal tail) with ^89^Zr/Gd^III^-LTL–PEG dissolved in saline at a dose of 6 MBq, and PET–CT scans were acquired at 4 min, 2 h, 18 h, 4 d, and 7 d. The images are displayed in SUVs, corrected for activity decay (Figure 5A–F), and the graphical quantification is presented for each organ of interest (Figure 5F). The in vivo biodistribution of the probe was evaluated by defining an ROI in the organs of interest (Figure 6A and Table S1). At the end of the last imaging time point (7 d), the mice were euthanized, the organs were harvested, and their Si-content was determined by ICP–OES (Table S2, post radioactive decay).

From the in vivo kinetic biodistribution profiles of the probe in the organs presented as a percentage of injected dose per volume of ROI, it can be seen that ^89^Zr/Gd^III^-LTL–PEG accumulates first in the lungs and moves slowly to the liver and bones over time (Figure 6A). The observed hepatobiliary uptake is characteristic for nanoparticles,^32,33^ which typically do not follow renal elimination due to their size larger than renal pores.^34^ The in vivo PET images do indeed show no renal uptake, as harvesting of the kidneys after 24 h p.i. and measuring Si-content by ICP–OES showed only 1.9 ± 0.2% ID/g (Table S3).

**Figure 7:**
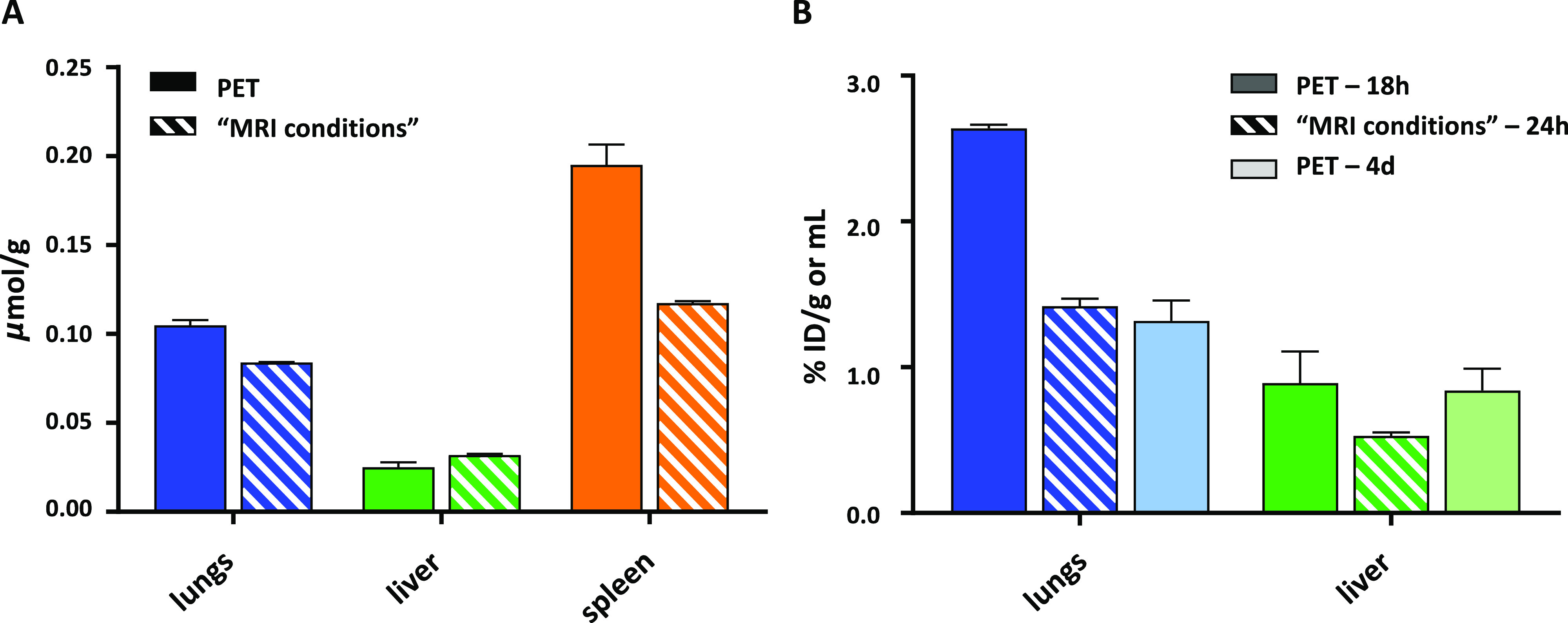
Comparison of organ uptake obtained from the PET and “MRI-conditions” studies: (A) data are calculated from ICP–OES and expressed in μmol Si/g, ex vivo PET data are taken at 7 d p.i., and ex vivo “MRI-conditions” data are taken at 24 h p.i.; (B) data are expressed in % ID per ROI volume (mL) for the in vivo PET data, at 18 h and 4 d p.i. time points, or per weight of organs (g) for the ex vivo “MRI-conditions” data at 24 h p.i.

Lung uptake is also often reported when evaluating nanoparticle distribution,^35^ including other zeolite-based probes,^36−38^ and is even proposed as promising for the pulmonary therapy.^39,40^ To further understand the observed lung accumulation of ^89^Zr/Gd^III^-LTL–PEG and foresee its MRI applicability, a parallel biodistribution study was performed by ex vivo ICP–OES (Figure 6B). It was hypothesized that injection of a higher concentration of the probe (≈10-fold higher for MRI than for PET) would trigger potential aggregation of the particles in vivo and therefore lead to a higher lung accumulation. A comparative graph, presented as μmol Si/g tissue and depicted in Figure 7A and Tables S2/S3, shows lung accumulation at 24 h p.i. under “MRI conditions” in line with the one observed at 7 d post PET study (ex vivo). These findings allow us to assume that nanoparticle’s aggregation is most likely not the reason for the increased lung accumulation. Such an upregulated lung affinity was associated with the porosity of nanoparticles, while size appeared to be important for the nonporous analogies. This was demonstrated for the mesoporous and solid SiO_2_ nanoparticles, with the affinity being attributed to the transient association with capillaries rather than internalization by the pulmonary cells.^41^

Finally, accumulation in spleen, also typically observed for nanoparticles,^27^ was evaluated. It is worth noting that an accurate definition of the ROI of spleen was rather difficult due to the strong overlap of the signals originating from the lungs and/or liver in the PET–CT images. Therefore, the actual spleen accumulation determined by PET was most likely included in the signals of the lungs and liver. However, for the ex vivo biodistribution studies upon the final imaging point (data analyzed by ICP–OES), the spleen, liver, and lungs could be collected separately, and their accurately determined uptake showed consistency in both experiments (Figures 7A).

Moreover, when plotting the percentage of injected dose per volume based on the in vivo PET images at 18 h and 4 d p.i., together with the % ID per weight of tissue at 24 h p.i. based on the ex vivo “MRI condition” study, we observe a consistent time-line distribution profile (Figure 7B). Importantly, when analyzing the Gd/Zr versus Si content within the organs evaluated, we can conclude that the particles are distributed as a whole, since the element ratio is consistent through the organs and with the expected ratio (see Table S3). This ratio is also consistent with the molar ratio calculated in the 150 μL solution injected, and it matches the expected “theoretical” Gd/Si ratio.

To exclude possible dissociation of ^89^Zr, the biodistribution profiles of ^89^Zr/Gd^III^-LTL–PEG were compared to those of ^89^ZrCl_4_ reported in the literature that show blood and bone accumulation from 1 h post intravenous injection (p.i.) in mice of about 55% ID/g and 15–20% ID/g, respectively.^36,42^ However, no blood uptake is detected for the ^89^Zr/Gd^III^-LTL–PEG, which is in agreement with the in vitro relaxivity studies (Figure 4) performed in the presence of serum showing no decrease in *r*_1_-relaxivity that would be expected in the case of pore blockage by bound proteins. This is also consistent with the radiochemical stability of the probe in different media (Figure S7), which indicates that the probe is reasonably stable up to 7 days, with only 6% leakage of ^89^Zr observed. Furthermore, the observed bone uptake of 1.8 ± 0.1% ID/mL at 2 h p.i. reaches ≈13% ID/mL at 4 d and stays constant up to 7 d. Even if there is a small leakage that could potentially account for the bone uptake observed, we cannot exclude the possibility of bone marrow uptake, also widely described for nanoparticles due to the higher leakage properties of the tissue, which contributes for the overall signal observed on the backbone. Both bone or bone marrow signals cannot be discerned in the PET images, and the inexistent or very low uptake in the remaining skeleton bones, mainly femur, upper limbs, and skull, is consistent with this observation, which in turn confirms good in vivo stability of the ^89^Zr/Gd^III^.

## 4 Conclusions

^89^Zr is a currently emerging radiotracer for PET imaging with characteristics convenient for tracking of biological processes with relatively long pharmacokinetics, typical for certain pathologies. In this study, we present a new type of probe for the rapid radiolabeling with ^89^Zr by exploiting the porous structure of LTL–zeolite nanoparticles, which have already been shown to exhibit versatile potential, including superior MRI properties. A high radiolabeling yield of Gd^III^-LTL was achieved using ^89^Zr-chloride. The commonly applied ^89^Zr-oxalate was demonstrated to be rather disadvantageous. Although positively charged Zr^IV^ ions (in chloride form) enter the negatively charged inner cavities of the zeolite, the large negatively charged Zr^IV^-oxalate complexes tend to accumulate on the surface, as evidenced by the significantly increased external surface area calculated from BET-analysis isotherms. It was also shown that the coloading of Zr^IV^ in addition to the already incorporated Gd^III^ inside the pores and decoration of the surface with PEG molecules (up to 6 wt %) did not affect both the *r*_1_- and *r*_2_-relaxivities, and therefore, the MRI properties of the probe could be preserved. Moreover, our straightforward radiolabeling protocol prevents in vivo metal leakage, while bypassing complicated Zr^IV^-complexation procedures.

The in vivo performance of the ^89^Zr/Gd^III^-LTL was investigated in healthy mice by recording PET-CT images over time after intravenous administration of the probe. The biodistribution profile showed initial lung uptake, followed by gradual migration of the probe to the liver and spleen. This behavior was found to be consistent with that of other nanoprobes reported in the literature. Interestingly, the prolonged in vivo retention of the ^89^Zr/Gd^III^-LTL–PEG allowed the evaluation of its biological stability, which appeared solid based on the ex vivo data, confirming the integrity of this multimodal imaging probe.

Finally, this study leads us to consider theranostic applications of the LTL-nanozeolite by including additional radiometals with therapeutic properties. The observed transient lung accumulation of the probe could provide interesting options to explore its pulmonary applications after extensive accumulation of the histological and biochemical data. It is in any case clear that further development of the presented PET/MRI probe would benefit from functionalization of the nanozeolite surface with vector molecules specific for biological targets associated with oncological pathologies.

## Supporting Information Available

Radiochromatograms; XRD patterns of the native and Zr/Gd-loaded LTL; photographs of Zr/Gd-loaded LTL suspended in various media overtime; DLS data of Zr/Gd-loaded LTL in various media measured overtime; leakage of Zr from PEGylated and non-PEGylated Zr(oxalate)/Gd–LTL challenged by EDTA; BET analysis; radiolabeling stability of ^89^Zr/Gd–LTL in PBS, saline, and serum; and biodistribution data (PDF)
