# Supplementary material for: On the Versatility of Nanozeolite Linde Type L for Biomedical Applications: Zirconium-89 Radiolabeling and In Vivo Positron Emission Tomography Study
Source: ACS Appl Mater Interfaces. 2022 Jul 13;14(29):32788–98. doi: 10.1021/acsami.2c03841 (PMC9335405; doi:10.1021/acsami.2c03841)
Supplement: Supplementary file 1 — am2c03841_si_001.pdf [file am2c03841_si_001.pdf]

Supporting Information

On the Versatility of Nanozeolite LTL for Biomedical Applications: Zirconium-89 Radiolabeling and *in vivo* PET study

Sara Lacerda[a], Wuyuan Zhang[b, †], Rafael T. M. de Rosales[c], Isidro Da Silva[d], Julien Sobilo[e], [5] Stéphanie Lerondel[e], Éva Tóth[a], Kristina Djanashvili[a,b,f]*

[a] Centre de Biophysique Moléculaire, CNRS UPR4301, Rue Charles Sadron, 45071 Orléans Cedex 2, France

[b] Department of Biotechnology, Delft University of Technology, Van der Maasweg 9, 2629 HZ, Delft, The Netherlands

[c] School of Biomedical Engineering & Imaging Sciences, King's College London, St Thomas' Hospital, London SE17EH, UK

[d] CEMHTI, CNRS UPR3079, Université d'Orléans, 45071 Orléans 2, France

[e] Centre d'Imagerie du petit Animal, PHENOMIN-TAAM, CNRS UAR44, F-45071 Orléans 2, France

[f] Le Studium, Loire Valley Institute for Advanced Studies, 1 Rue Dupanloup, 45000 Orléans, France

[†] Present Address: Tianjin Institute of Industrial Biotechnology, Chinese Academy of Sciences, 300308 Tianjin, China

*Corresponding author: k.djanashvili@tudelft.nl

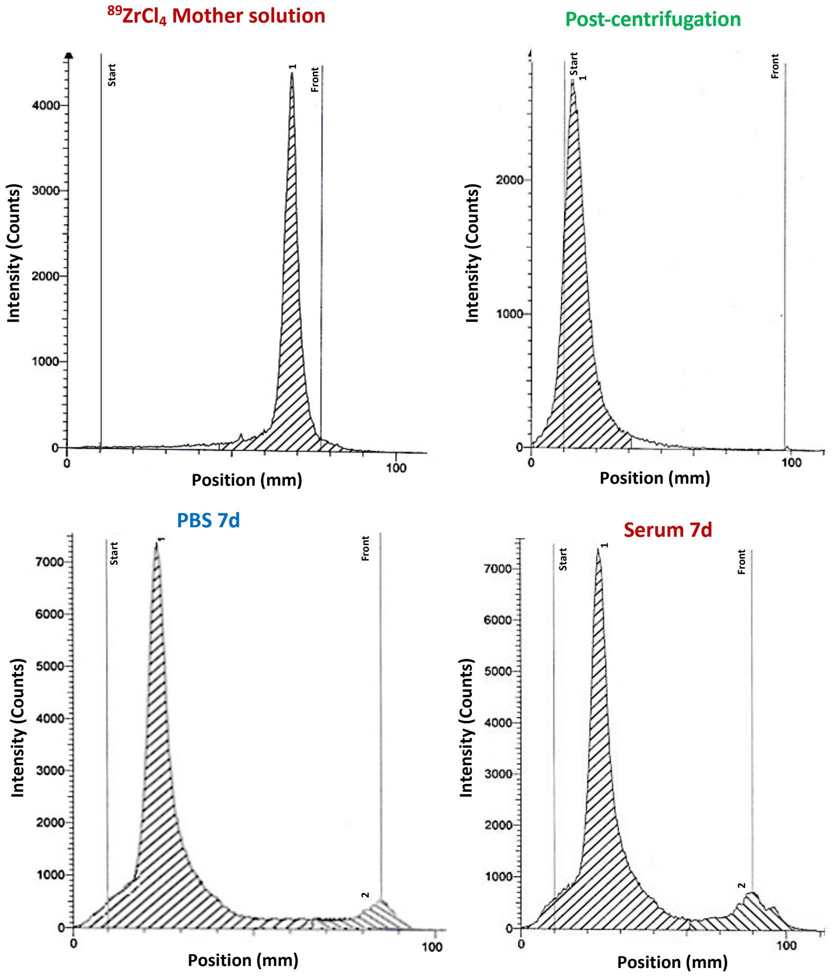

Figure S1. Radio-chromatograms obtained for $^{89}ZrCl_4$ solution, ^{89}Zr-Gd^{III}-LTL resuspended in saline, and ^{89}Zr-Gd^{III}-LTL-PEG incubated in PBS and serum for 7 days.

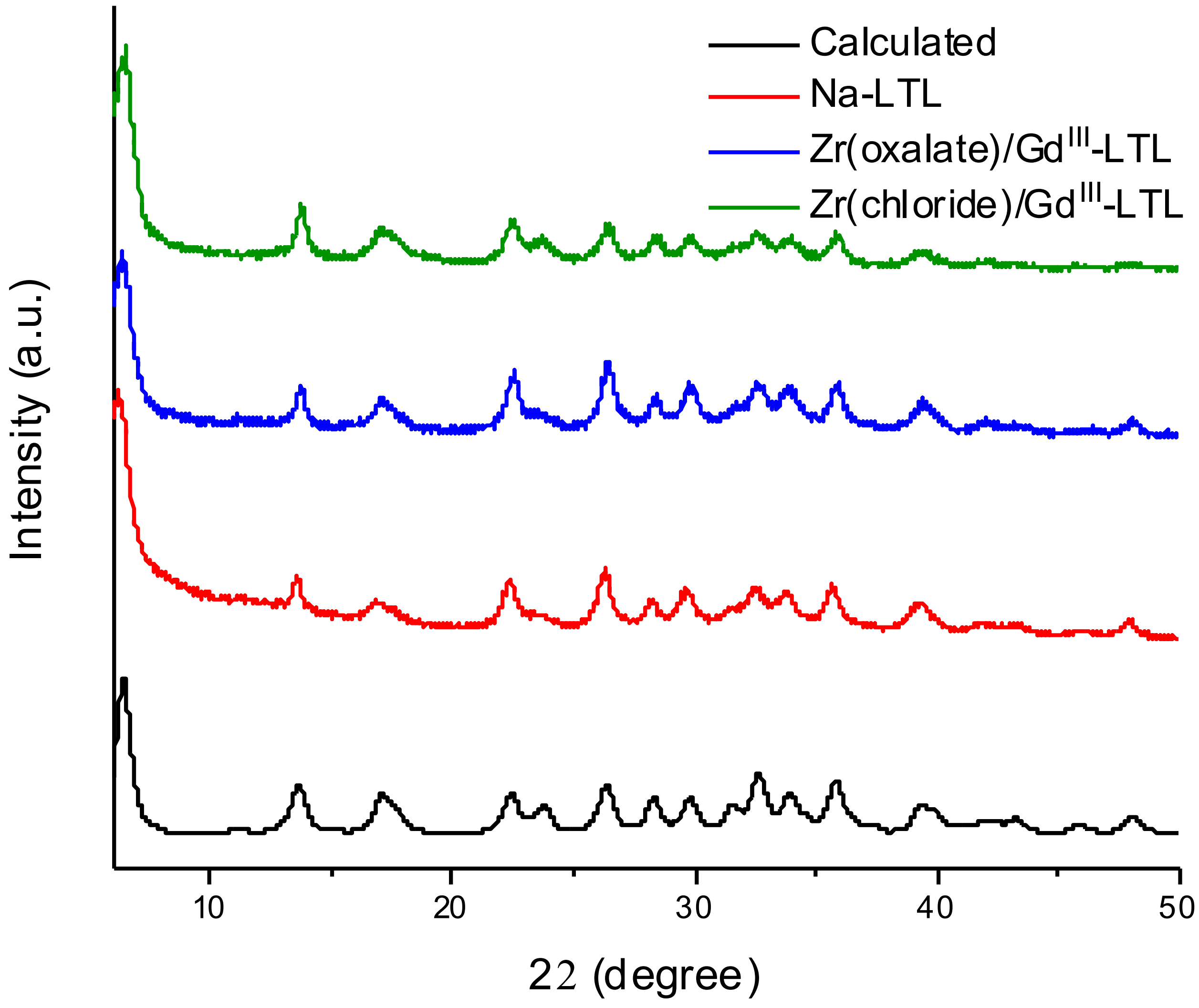

Figure S2. XRD profiles of LTL-crystals before (Na-LTL, red line) and after loading with Gd^{III} and Zr^{IV} via oxalate (blue line) and chloride (green line) procedures. The profile presented by the black line corresponds to the calculated pattern from the database of International Zeolite Association of zeolite structures for the Linde Type LTL framework (http://www.iza-online.org/, 2020). The patterns were obtained using a Bruker AXS/D8 Advance diffractometer equipped with a Lynxeye detector and Co Kα radiation (λ = 1.78897 Å, 35 kV, 40 mA). The measurement range was from 5 to 70 °2θ with a step size of 0.02° in continuous mode and an acquisition time of 0.5 s per step, repeating the scanning until a good signal-to-noise ratio was achieved.

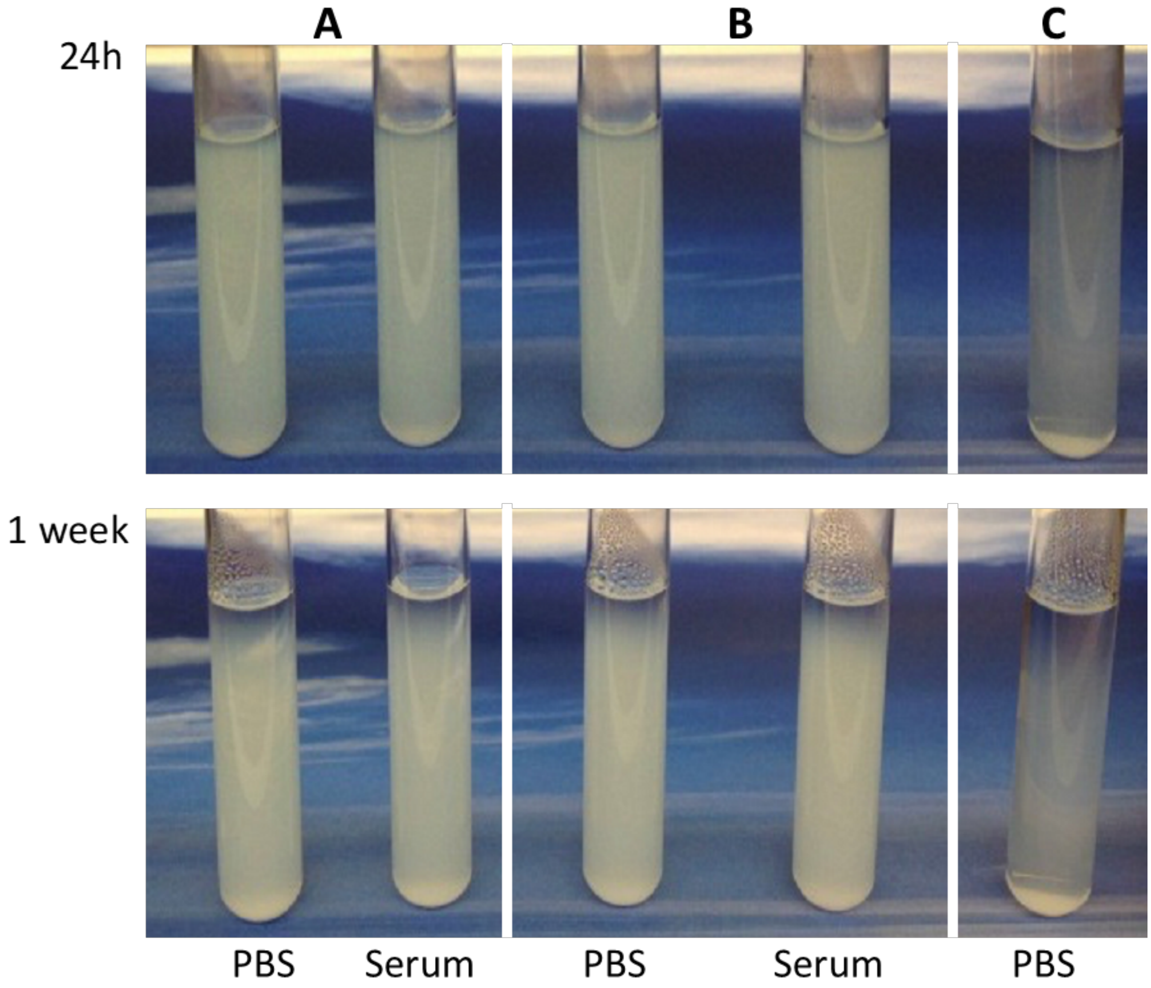

Figure S3. Visual observations of LTL-nanoparticles suspended at a concentration of 500 μg/mL in PBS and serum for 24 hours and 1 week at 37°: **A**) Zr/(oxalate)Gd^{III}-LTL-PEG, **B**) Zr/(chloride)Gd^{III}-LTL-PEG, and **C**) Pre-loaded Na^{I}-LTL-nonPEGylated.

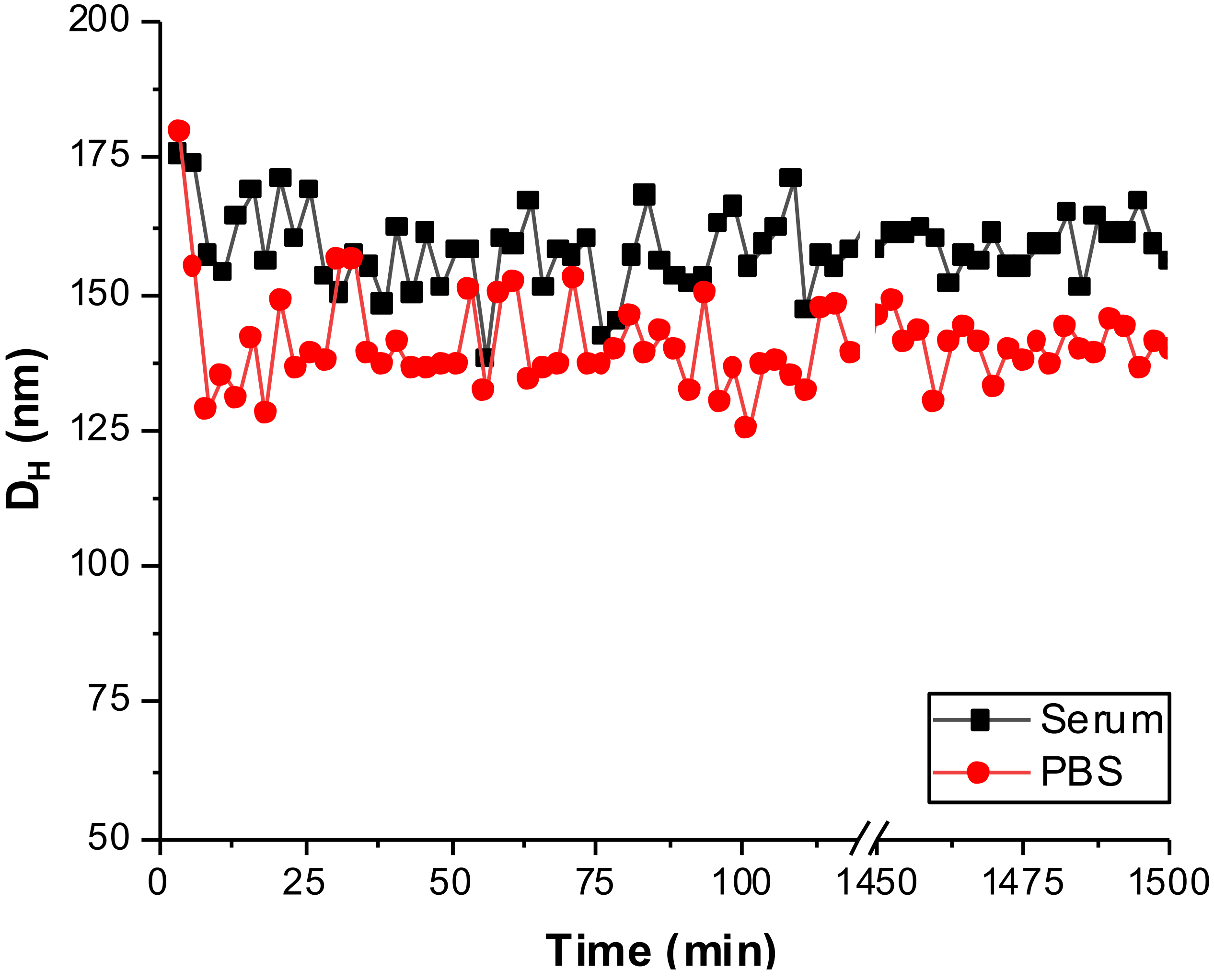

Figure S4. Colloidal stability of Zr(chloride)/Gd^{III}-LTL-PEG suspended at a concentration of 500 μg/mL in PBS and serum and measured over time by dynamic light scattering (DLS).

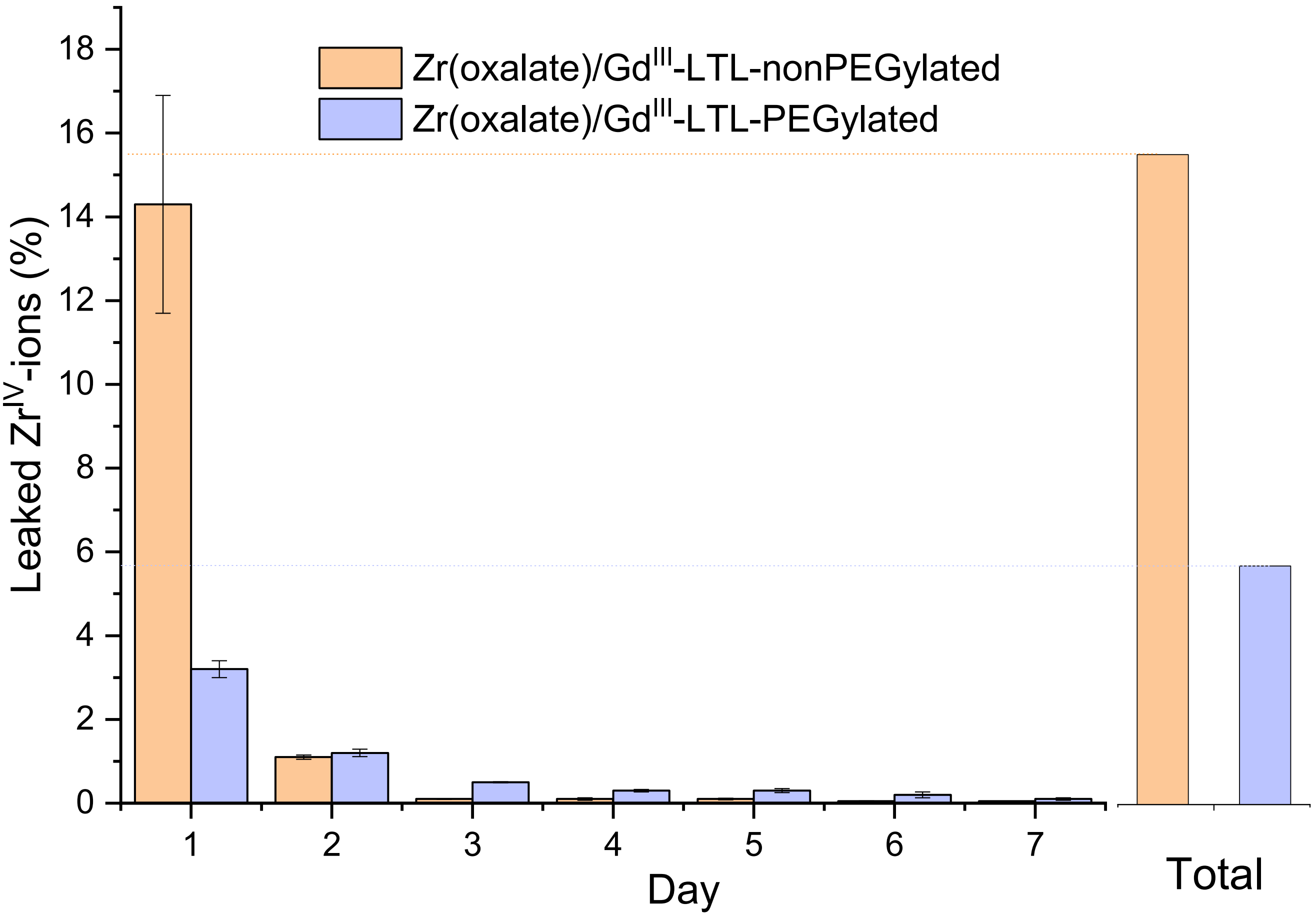

Figure S5. Leakage of Zr^{IV} ions from nonPEGylated and PEGylated Zr(oxalate)/Gd^{III}-LTL incubated at 37 °C in 1 mM solution of EDTA for 7 days. The samples were centrifuged, the Zr-content in the supernatant was measured by the ICP-OES, the tubes were refilled with again with EDTA-containing medium and kept until the next measurement.

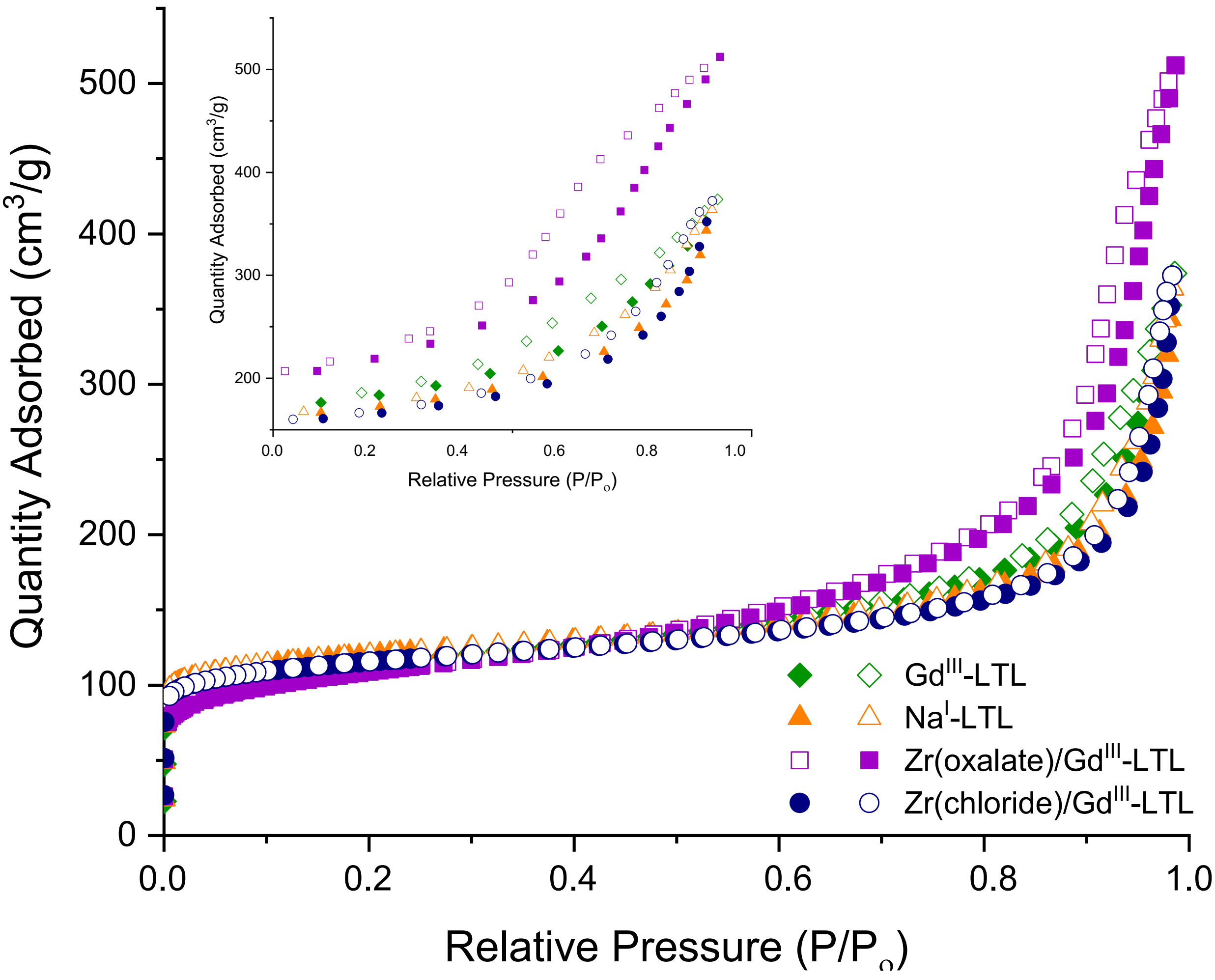

Figure S6. Nitrogen adsorption and desorption isotherms of LTL before (Na^{I}) and after the loading with extra-framework Gd^{III} and Zr^{IV} via both, oxalate and chloride procedures. The hysteresis at high P/P_O is visualized in the inset.

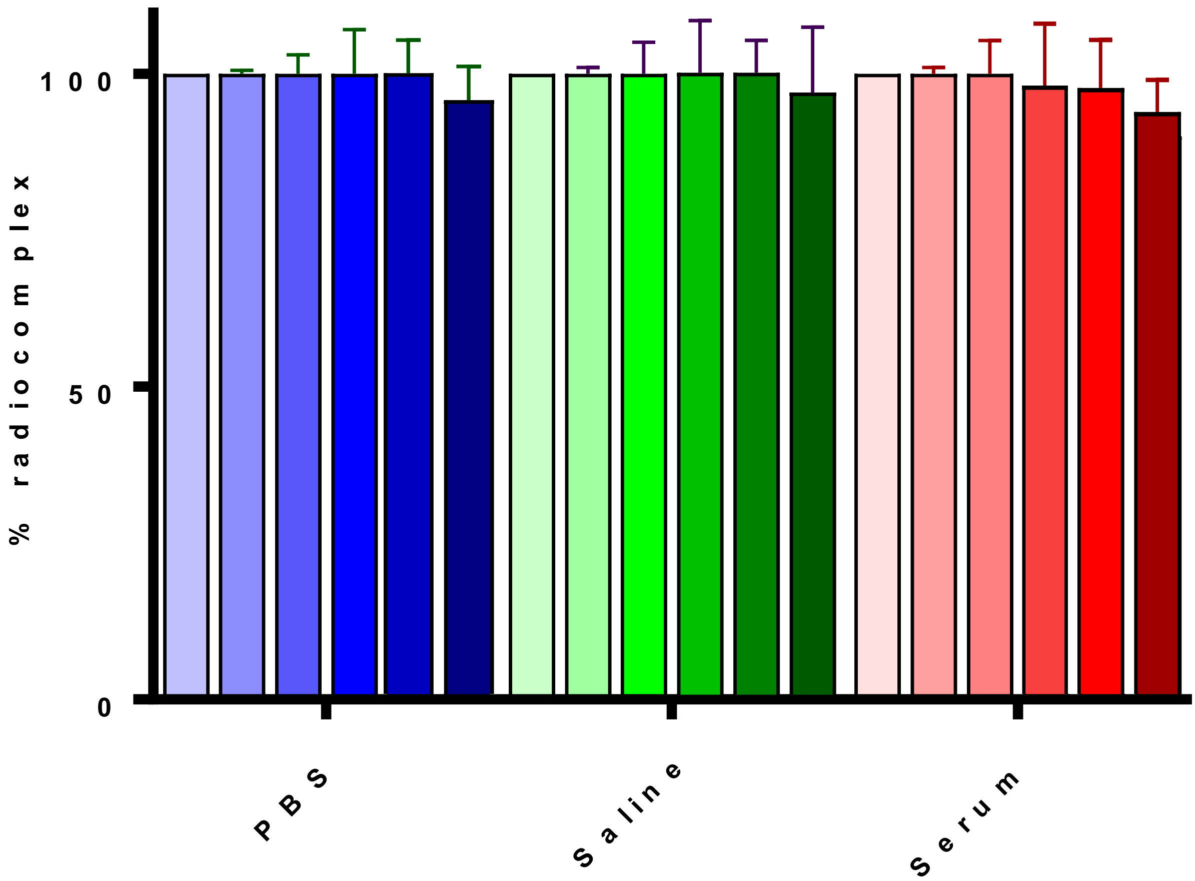

Figure S7. In vitro stability of ^{89}Zr-Gd^{III}-LTL in different media (PBS, saline (0.9% NaCl) and serum) at different time points: 0, 0.3, 1, 2, 3 and 7 days (lighter to darker color, respectively).

Table S1. Biodistribution based on *in vivo* PET images.

Time p.i.	Lungs	Liver (%ID/mL ± SEM)	Bones
4 min	41.3 ± 3.9	7.8 ± 1.3	0
2 h	34.8 ± 2.5	8.0 ± 0.5	1.4 ± 0.6
18 h	26.4 ± 0.4	8.9 ± 3.8	7.1 ± 1.4
4 d	13.2 ± 2.4	8.4 ± 2.6	12.9 ± 1.5
7 d	9.9 ± 2.6	8.2 ± 2.2	12.8 ± 0.4

Table S2. Biodistribution based on *ex vivo* "PET conditions" study at 7 d p.i.

Organ	Si (µmol/g ± SEM)
lungs	0.21 ± 0.01
liver	0.05 ± 0.01
bone	0.30 ± 0.11
spleen	0.39 ± 0.04
muscle	0.39 ± 0.20

Table S3. Biodistribution based on *ex vivo* "MRI-conditions" study at 24 h p.i.

Organ	Gd	Si	Gd / Si ratio[a]	Gd	Si
	(µmol/g ± SEM)			(%ID/g ± SEM)	
injected solution[b]	2.56 x 10^{-7} mol	1.21 x 10^{-6} mol	0.21	-	-
lungs	0.036 ± 0.002	0.168 ± 0.001	0.22	14.2 ± 0.9	13.9 ± 0.1
liver	0.013 ± 0.001	0.064 ± 0.002	0.21	5.3 ± 0.4	5.3 ± 0.1
spleen	0.049 ± 0.001	0.235 ± 0.003	0.21	19.3 ± 0.5	19.4 ± 0.2
kidneys	0.005 ± 0.0003	0.023 ± 0.002	0.21	1.9 ± 0.1	1.9 ± 0.2

[a)] Theoretical Gd/Si ratio for the loading of Gd of 2.7 wt% is 0.22. The value is derived from the general formula of the LTL zeolite: $K_6Na_3(H_2O)_{21}Al_9Si_{27}O_{72}$ taking into account that per cage 3 Na^{I}-ions can be exchanged with 1 Gd^{III}-ion.
[b)] Gd and Si content (in mol) in the 150 µL injected *in vivo*, calculated by ICP-OES.
